# *MKL1* expressed in macrophages contributes to the development of murine colitis

**DOI:** 10.1038/s41598-017-13629-0

**Published:** 2017-10-20

**Authors:** Jianbo An, Takashi Nagaishi, Taro Watabe, Taeko K. Naruse, Mamoru Watanabe, Akinori Kimura

**Affiliations:** 1 Department of Molecular Pathogenesis, Medical Research Institute, Tokyo Medical and Dental University (TMDU), Tokyo, Japan; 2 Department of Gastroenterology and Hepatology, Graduate School of Medical Science, Tokyo Medical and Dental University (TMDU), Tokyo, Japan

## Abstract

Mice deficient in the megakaryoblastic leukaemia 1 (*Mkl1)* gene experience less severe dextran sulphate sodium (DSS)-induced colitis, implying that *Mkl1* plays a pathological role in inflammatory bowel disease (IBD). However, the contribution of *Mkl1* to the development of colitis remains to be elucidated. The expression of *Mkl1* is higher in the colonic lamina propria macrophages (LPMac) of DSS-treated mice than in those of control mice. Therefore, we established a transgenic mouse line that overexpresses human *MKL1* (MKL1-Tg) specifically in cells of the monocyte/macrophage lineage, in order to investigate the potential role of macrophage *MKL1* in the pathogenesis of colitis. MKL1-Tg mice displayed spontaneous colon shortening and rectal prolapse. Flow cytometric and quantitative RT-PCR analyses revealed that, in MKL1-Tg mice compared to littermate controls, the population of LPMac was decreased and had an altered inflammatory phenotype indicative of impaired anti-inflammatory properties, whereas bone marrow-derived macrophages from MKL1-Tg mice skewed towards M1 polarisation. In addition, MKL1-Tg mice had higher susceptibility to DSS-induced colitis than their littermate controls. These observations indicated that MKL1 crucially contributes to the development of colitis via the regulation of the function of macrophages, suggesting that it may be a potential therapeutic target for the prevention of IBD.

## Introduction

Inflammatory bowel disease (IBD), including Crohn’s disease and ulcerative colitis, is characterised by chronic, progressive inflammation of the gastrointestinal tract. IBD arises from immune dysfunction, in which aberrant immune responses evoke excessive inflammation^1,2^. It is widely accepted that both genetic and environmental factors are involved in the pathogenesis of IBD^1,3^; however, the precise aetiology is still not well understood.

Megakaryoblastic leukaemia 1 (MKL1), also called myocardin-related transcription factor A, is an actin-binding protein that shuttles between the cytoplasm and nucleus in response to Rho–Rock signalling^4^. In the nucleus, MKL1 mediates various cellular functions as a co-activator of serum response factor. For example, MKL1 plays a key role in the regulation of the proliferation and migration of vascular smooth muscle cells^5^. The functions of MKL1 in leukocytes have been intensively investigated. The loss of MKL1 in neutrophils results in the disruption of actin filaments and impaired phagocytosis, and a patient with homozygous nonsense mutations in *MKL1* failed to efficiently clear bacteria^6^. In macrophages, MKL1 mediates the upregulation of pro-inflammatory cytokines in response to inflammatory stimuli^7^. Interestingly, murine experimental colitis is less severe in *Mkl1*-knockout mice^7^, suggesting the involvement of *Mkl1* in IBD. Given that the immune system plays a central role in IBD, it is likely that MKL1 in leukocytes contributes to the development of colitis.

Macrophages constitute the most abundant population in the intestinal lamina propria. Through frequent interactions with the enteric microflora and crosstalk with the adaptive immune system, lamina propria macrophages (LPMac) govern the immune network, which is indispensable for gut defence and homeostasis^8^. Recent studies have indicated that LPMac derived from Ly6C^+^ blood monocytes are highly ‘educated’; despite being phagocytic, they are unresponsive to inflammatory stimuli and generally display anti-inflammatory properties^8,9^. Collapses of gut homeostasis are often associated with alternations in the inflammatory phenotypes of LPMac^10,11^, a hallmark of IBD.

In this study, we investigated the involvement of MKL1 in IBD. We found that the expression of *Mkl1* was significantly elevated in LPMac, but not in whole colon tissue, in the setting of murine experimental colitis, compared to that in the steady state, suggesting an important role for MKL1 in macrophages in the development of IBD. We established a line of transgenic mice, which overexpress human *MKL1* specifically in monocyte/macrophage lineage cells (MKL1-Tg). We found that LPMac from MKL1-Tg mice had a perturbed inflammatory phenotype, and that dextran sulphate sodium (DSS)-induced colitis in MKL1-Tg mice was fulminant compared to that in littermate controls. In addition, we found that *MKL1* overexpression was associated with skewed macrophage polarisation, which may underlie the molecular pathogenesis of IBD in MKL1-Tg mice.

## Results

### Elevated expression of *Mkl1* in colonic LPMac in experimental colitis

**Figure 1:**
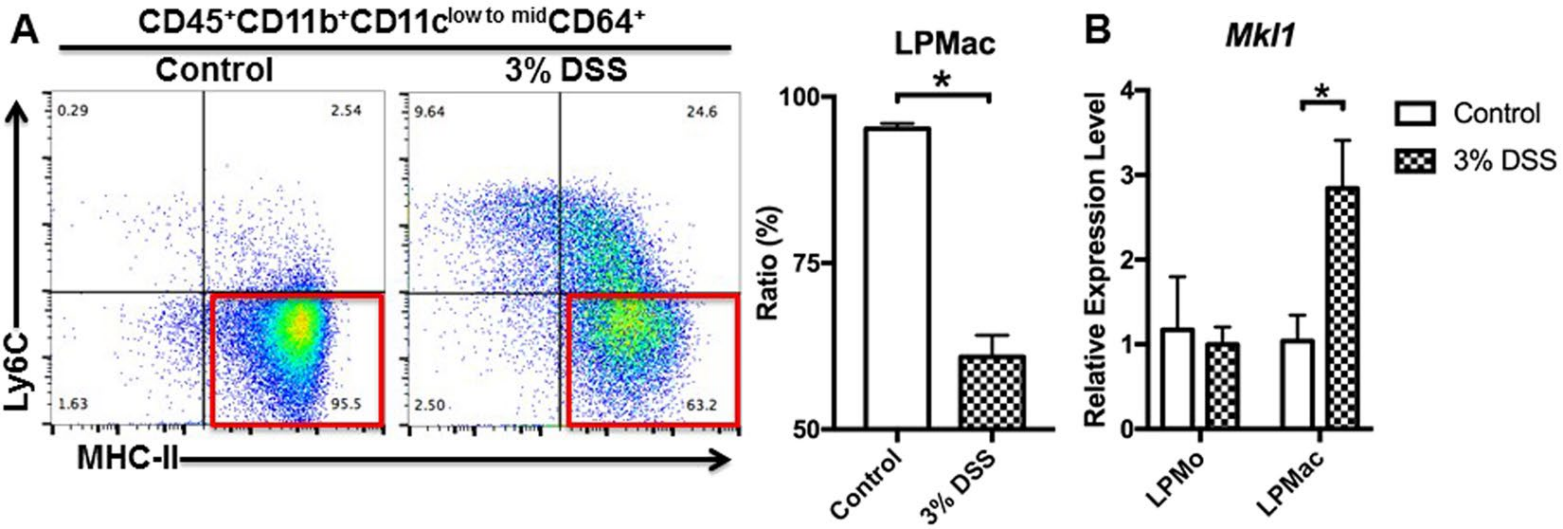
Perturbation of the LPMac phenotype was associated with upregulation of *Mkl1* during experimental murine colitis. C57BL/6 J mice were treated with 3% DSS or control drinking water for 4 days. (**A**) Representative cytograms of Ly6C and MHC-II expression on CD45^+^CD11b^+^CD11c^low to mid^CD64^+^ cells. We calculated the proportion (rate, %) of the cells in the red frames, designated LPMac. (**B**) The expression levels of *Mkl1* in CD45^+^CD11b^+^CD11c^low to mid^ CD64^+^Ly6C^+^ LPMo and CD45^+^CD11b^+^CD11c^low to mid^ CD64^+^Ly6C^—^MHC-II^+^ LPMac were determined by quantitative RT-PCR. All experiments were repeated 5 times and data are shown as mean ± standard deviation (SD). *P < 0.05.

We analysed the phenotypes of colonic LPMac by flow cytometry, using antibodies against CD45, CD11b, CD11c, CD64, Ly6C, and MHC-II^10,11^. We observed that mice that received 3% DSS had a decreased proportion of LPMac accompanied by a significant increase in the proportion of lamina propria monocytes (LPMo) (Fig. 1A and Supplementary Fig. S1). We next investigated the expression of *Mkl1* in colonic tissues. In the steady state, isolated LPMo and LPMac showed comparable expression of *Mkl1* (Fig. 1B). However, LPMac, but not LPMo, had increased expression of *Mkl1* in DSS-treated mice (Fig. 1B). In addition, the expression of *Mkl1* was unchanged in the colons of control and DSS-treated mice (Supplementary Fig. S2). The upregulation of *Mkl1* in LPMac was associated with decreased expression of *Pparg* (Supplementary Fig. S3). These observations suggested a potential role of macrophage *Mkl1* in the pathogenesis of experimental colitis.

### Establishment of MKL1-Tg mice

**Figure 2:**
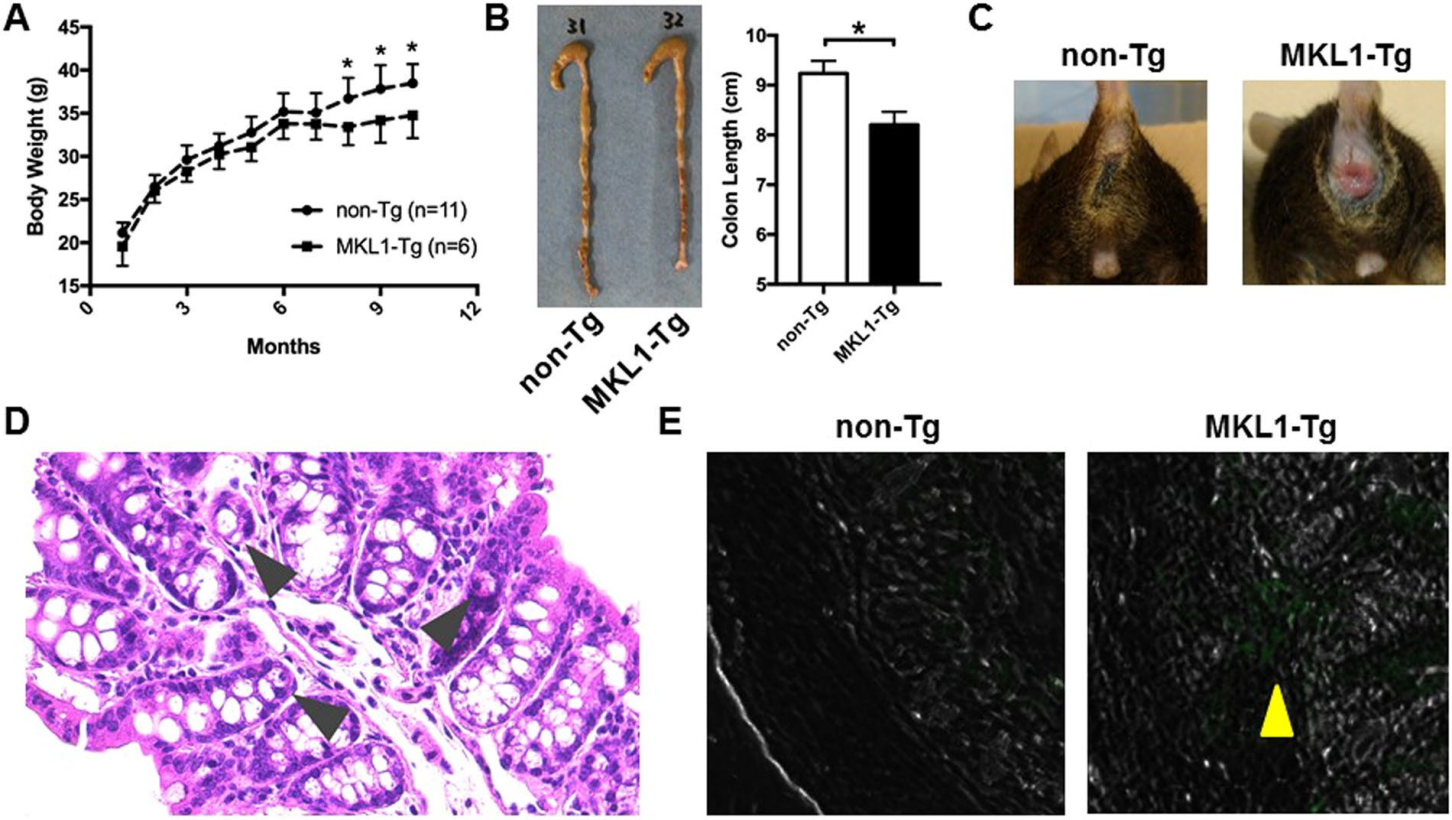
Characterisation of MKL1-Tg mice. (**A**) Body weights of male MKL1-Tg mice and non-Tg littermates were measured monthly. (**B**) Representative photograph of whole colon from female MKL1-Tg mice and control littermates at 4 months after birth. Colon length was measured (n = 3 for each group). Data are shown as mean ± SD. *P < 0.05. (**C**) Representative photographs of the perianal region of male control and MKL1-Tg mice with rectal prolapse. (**D**) Representative histological image showing cryptitis (arrowheads) in H&E-stained colonic tissue of 8-month-old MKL1-Tg mice. (**E**) Representative immunohistochemical images showing CD68^+^ cells stained with rat anti-CD68 antibody and Alexa Fluor® 488-conjugated goat anti-rat IgG. Green cells represent CD68^+^ cells (arrowhead) infiltrating the colonic submucosal layer of MKL1-Tg mice.

We established a transgenic mouse line that overexpresses human *MKL1* specifically in monocytes/macrophages, driven by the human *CD68* promoter element (Supplementary Fig. S4A and B). Compared with bone marrow monocytes, bone marrow-derived macrophages (BMDMs) from MKL1-Tg mice expressed higher levels of the transgene, *MKL1* (Supplementary Fig. S4C). We found that MKL1-Tg mice had lower body weights than their non-Tg littermates, which reached statistical significance approximately 8 months after birth (Fig. 2A). At approximately 4 months of age, MKL1-Tg mice showed spontaneous shortening of the colon (Fig. 2B) and occasionally developed rectal prolapse (Fig. 2C). Histological analysis revealed scattered cryptitis in the colonic epithelial layer of MKL1-Tg mice (Fig. 2D). In addition, we observed infiltration of CD68^+^ cells into the colonic submucosal layer of MKL1-Tg mice (Fig. 2E).

### LPMac from MKL1-Tg mice had aberrant inflammatory phenotypes

**Figure 3:**
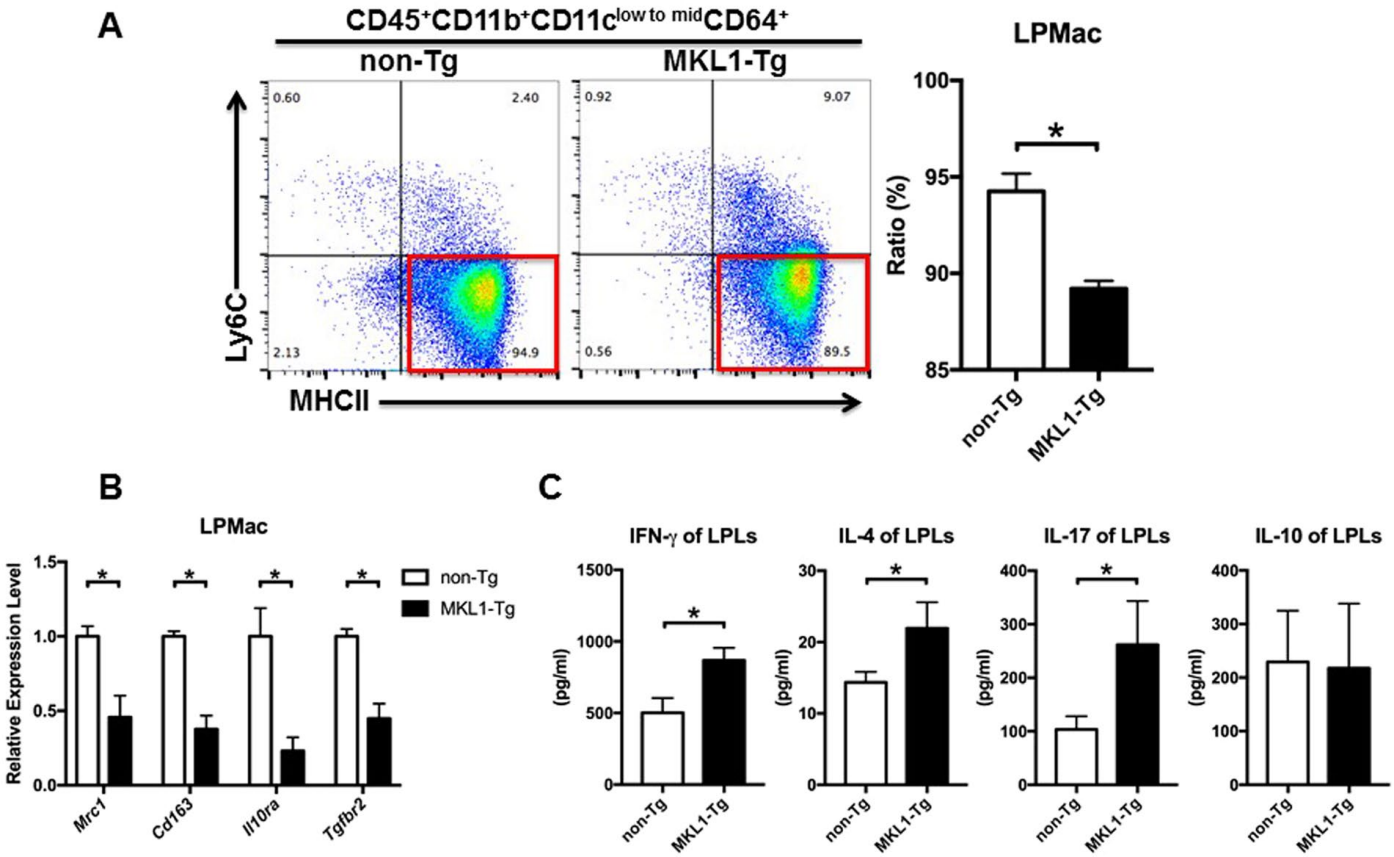
*MKL1* overexpression affected the inflammatory phenotype of LPMac. (**A**) Representative cytograms showing the Ly6C and MHC-II expression on CD45^+^CD11b^+^CD11c^low to mid^CD64^+^ cells from 6-month-old MKL1-Tg and non-Tg littermates. We also calculated the proportions of LPMac. (**B**) The expression levels of *Mrc1*, *Cd163*, *Il10ra*, and *Tgfbr2* in CD45^+^CD11b^+^CD11c^low to mid^CD64^+^Ly6C^—^MHC-II^+^ LPMac were determined by quantitative RT-PCR. (**C**) The concentration of IFN-γ, IL-4, IL-17, and IL-10 in supernatants from LPLs were measured by ELISA. All experiments were repeated 3 times and data are shown as mean ± SD. *P < 0.05.

Flow cytometric analysis showed that MKL1-Tg mice had more LPMo and fewer LPMac than control littermates (Fig. 3A and Supplementary Fig. S5). To further investigate the perturbation of LPMac in MKL1-Tg mice, we isolated mRNA from LPMac and performed quantitative RT-PCR. We found that the expression of the tissue-resident macrophages markers *Mrc1* and *Cd163*, and the receptors for the anti-inflammatory cytokines IL-10 and TGF-β, were lower in MKL1-Tg mice than in control littermates (Fig. 3B). In addition, the expression of *Pparg* was significantly decreased in LPMac from MKL1-Tg mice (Supplementary Fig. S6). Furthermore, cultured colonic lamina propria lymphocytes (LPLs) from MKL1-Tg mice produced more IFN-γ, IL-4, and IL-17 than those from non-Tg littermates (Fig. 3C).

### MKL1 orchestrated macrophage polarisation

**Figure 4:**
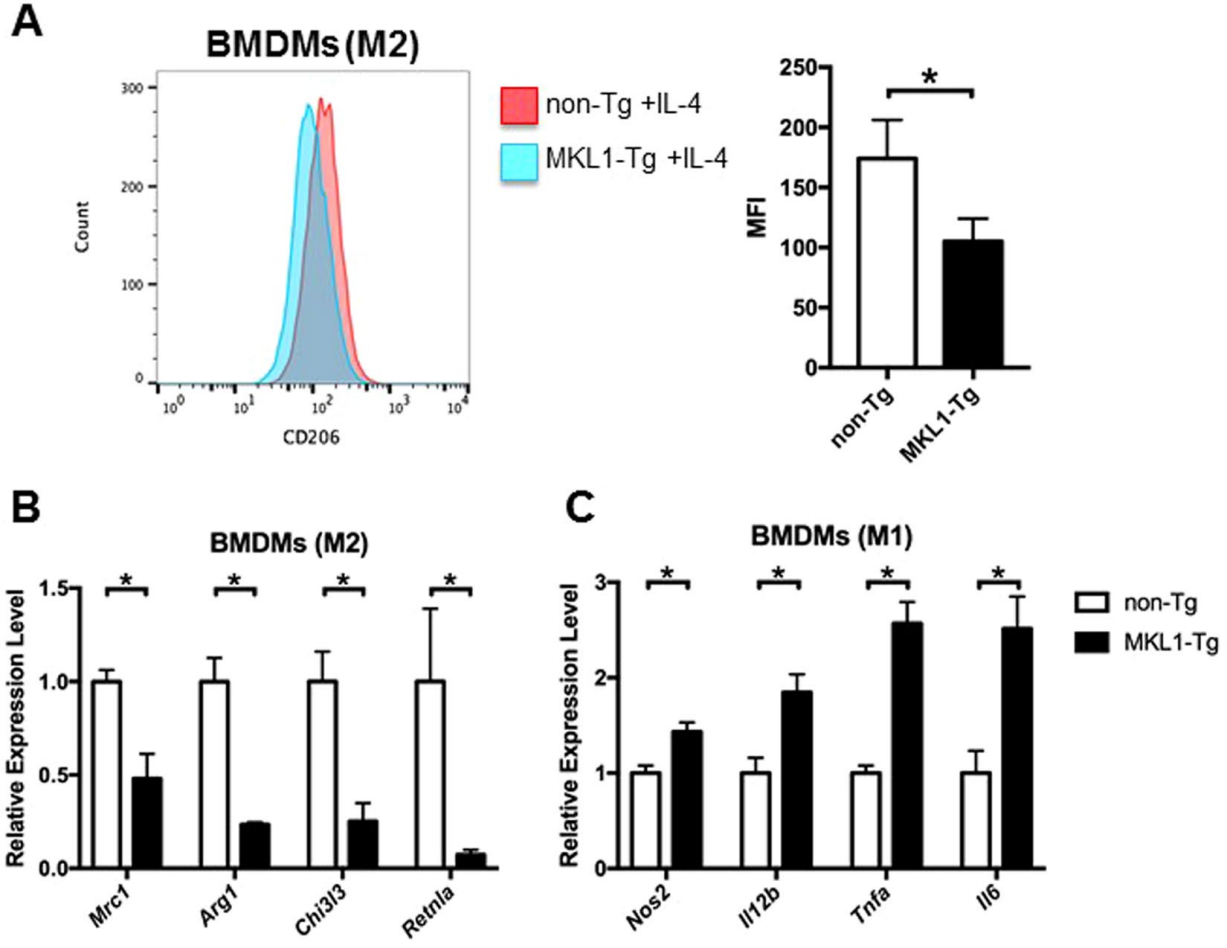
*MKL1* modulated macrophage polarisation. (**A**) BMDMs from MKL1-Tg mice and non-Tg littermates were treated with rIL-4 (20 ng/ml) for 24 hours. The expression of CD206 on CD11b^+^F4/80^+^ BMDMs (histograms) was evaluated by flow cytometry. We calculated and plotted the mean fluorescence intensity (MFI). BMDMs from MKL1-Tg mice and non-Tg littermates were either treated with rIL-4 (20 ng/ml), or rIFN-γ (100 ng/ml) + LPS (50 ng/ml) for 16 hours. The expression levels of (**B**) M2 markers *Mrc1*, *Arg1*, *Chi3l3*, and *Retnla*, and (**C**) M1 markers *Nos2*, *Il12b*, *Tnfa*, and *Il6* were determined by quantitative RT-PCR. All experiments were repeated 3 times and data are shown as mean ± SD. *P < 0.05.

Treatment with IFN-γ+ lipopolysaccharide (LPS) or IL-4 *in vitro* polarises macrophages into classically activated (M1) or alternatively activated (M2) types, respectively. It is known that the transcriptional activity of MKL1 is regulated by its nuclear localisation, which depends on actin dynamics^4^. We observed that, in BMDMs, expression of ectopic MKL1 was markedly increased in nuclei (Supplementary Fig. S7A); this was associated with increased formation of cytoplasmic filamentous actin (F-actin) (Supplementary Fig. S7B), and led to downregulation of *Pparg* (Supplementary Fig. 7C). Given that PPARγ regulates the polarisation of M2 macrophages^12^, we hypothesised that *MKL1* overexpression would affect macrophage polarisation. Using flow cytometric analysis, we demonstrated that the expression of the M2 marker CD206 was suppressed both in vehicle-treated and IL-4-treated BMDMs from MKL1-Tg mice, compared to its expression in non-Tg controls (Fig. 4A and Supplementary Fig. S8). Quantitative RT-PCR analysis showed impaired expression of the M2 marker genes *Mrc1*, *Arg1*, *Chi3l3*, and *Retnla*, in M2-polarised BMDMs from MKL1-Tg mice (Fig. 4B). On the other hand, upon LPS stimulation, we detected significantly higher NF-κB p65 transcriptional activity in nuclear extracts of BMDMs from MKL1-Tg mice than in those from control mice (Supplementary Fig. 7D). Furthermore, expression of *MKL1* promoted M1 polarisation, as indicated by increased expression of the pro-inflammatory genes *Nos2*, *Il12b*, *Tnfa*, and *Il6* in MKL1-Tg mice (Fig. 4C).

### DSS-induced colitis is fulminant in MKL1-Tg mice

**Figure 5:**
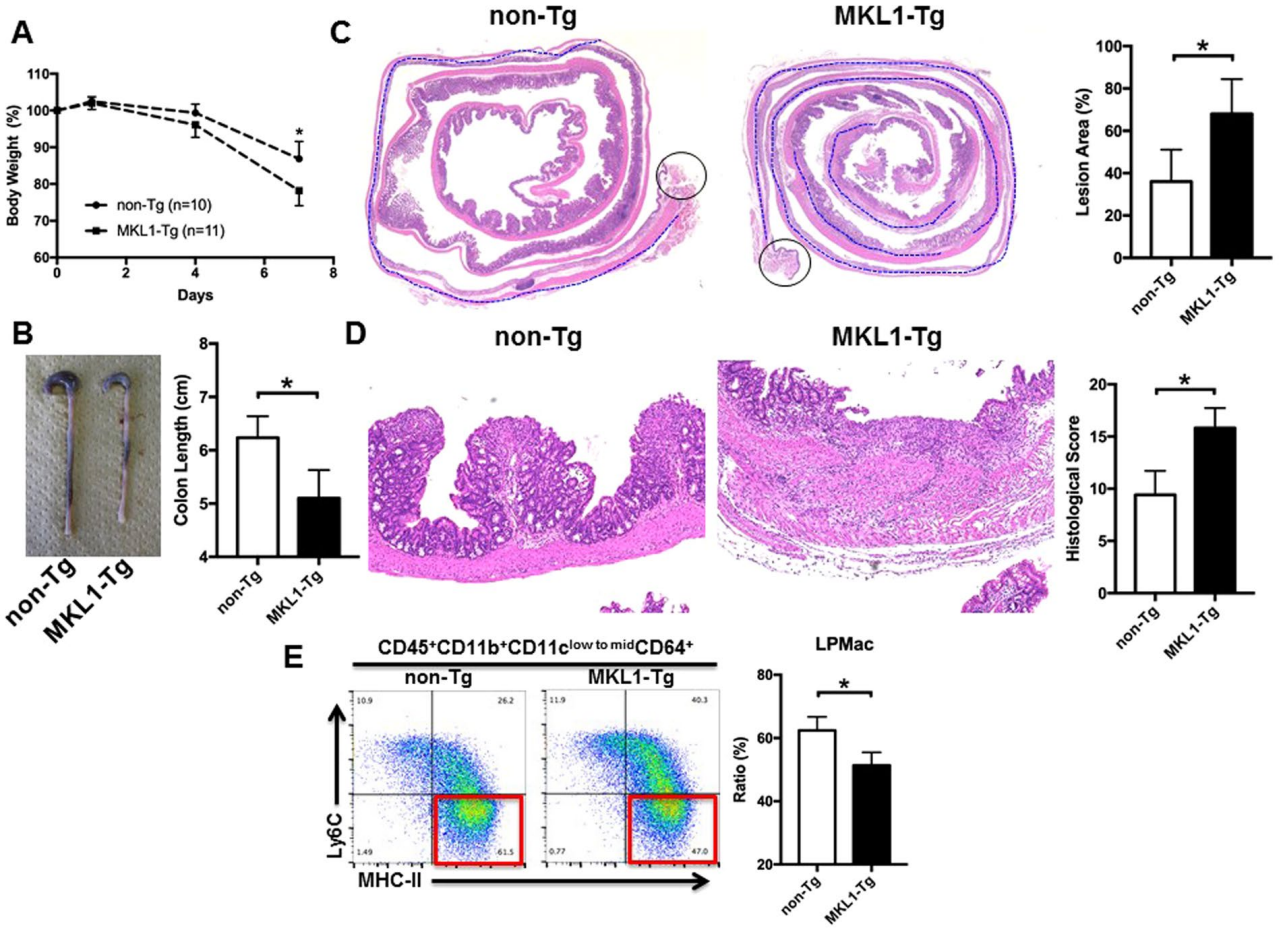
MKL1-Tg mice developed fulminant DSS-induced colitis. MKL1-Tg mice and non-Tg littermates (8 weeks old) received 3% DSS in their drinking water for 7 days. (**A**) Male body weights were measured on day 0, 1, 4, and 7, and are shown as the percentage of initial body weight. (**B**) Representative image of whole colons from female MKL1-Tg mice and control littermates. Colon length was measured (n = 3 for each group). (**C**) Representative histological images (low-power field) of Swiss-rolled whole colon stained with H&E. Regions specified with blue lines were affected by colitis. Positions of anuses are indicated by circles. Lesion area was measured and plotted (n = 5 for each group). (**D**) Representative histological images (high-power field) of the proximal region of the colon stained with H&E. We calculated the histological score (n = 5 for each group). (**E**) MKL1-Tg mice and non-Tg littermates received 3% DSS in the drinking water for 4 days. Representative cytograms showing Ly6C and MHC-II expression on the CD45^+^CD11b^+^CD11c^low to mid^ CD64^+^ cells. We calculated the proportions of LPMac. Experiments were repeated 3 times. Data are shown as mean ± SD. *P < 0.05.

As MKL1-Tg mice did not show spontaneous inflammation in the colonic mucosa until 8 weeks of age (Supplementary Fig. S9), we examined their susceptibility to DSS-induced colitis at this age. Compared to control littermates, MKL1-Tg mice showed more prominent weight loss (Fig. 5A) and shortening of the colon (Fig. 5B) after administration of 3% DSS for 7 days. In addition, histopathological analyses indicated that lesions of immune infiltration and ulceration extended from the proximal to the distal region of the colon in MKL1-Tg mice, whereas control littermates showed similar, but relatively mild lesions, mainly in the distal colon (Fig. 5C). Detailed histological assessment revealed that MKL1-Tg mice experienced more damaging colitis, especially of the proximal colon, as evidenced by the massive infiltration and penetration of inflammatory cells into the colonic tissues, including the lamina propria, submucosa, and muscle layers, and the destruction of the epithelial structure, accompanied by ulceration (Fig. 5D). Notably, the LPMac compartment was further decreased in MKL1-Tg mice upon treatment with 3% DSS (Fig. 5E and Supplementary Fig. S10).

In order to evaluate the histopathological features of the recovery, rather than the acute, phase of DSS-induced colitis, we induced colitis by a modified protocol. We administered 3% DSS for 5 days, followed by regular water for another 3 days (Supplementary Fig. S11A). In the colitis recovery phase, MKL1-Tg mice still experienced more prominent colon shortening (Supplementary Fig. S11B). Immunohistochemical analysis revealed prominent B cell infiltration and/or follicle formation in the colonic mucosa of MKL1-Tg mice, compared to that in control littermates, during the recovery phase (Supplementary Fig. S11C).

## Discussion

IBD is globally focused because of the difficulty in understanding its complex aetiology. Murine DSS-induced colitis is a classical model for studying the pathogenesis of IBD^13^, and *Mkl1*-knockout mice are known to develop DSS-induced colitis of mitigated severity^7^. We observed elevated expression of *Mkl1* in LPMac, but not in LPMo or the colon tissue, in DSS-treated mice. Given that MKL1 is involved in the regulation of macrophage activation^7^, we hypothesised that MKL1 in LPMac could modulate intestinal inflammation and, hence, contribute to the development of IBD.

CD68, or macrosialin in the mouse, is one of the canonical molecular markers of macrophages. Expression of *CD68* increases substantially during the differentiation of monocytes into macrophages^14^. In order to investigate the role of *MKL1* in LPMac *in vivo*, we selected the *CD68* promoter element, which was reported to effectively drive transgene expression in macrophages, but not in dendritic cells, lymphocytes, or granulocytes, in the colon^15^, to generate an MKL1-Tg line. The human *MKL1* transgene was successfully overexpressed in macrophages from MKL1-Tg mice. The transgene was also expressed in monocytes. However, its expression was much higher in BMDMs, indicating that the *CD68* promoter was largely activated during differentiation into macrophages. We observed that MKL1-Tg mice had a decreased proportion of colonic LPMac and a reciprocally increased LPMo population, compared to non-Tg controls. Perturbation of LPMac phenotypes has been reported to affect the function of effector T and/or regulatory T cells in murine colitis models^11,16^, and we detected increased production of IFN-γ, IL-4, and IL-17 by LPLs from MKL1-Tg mice compared to those from controls, indicating enhanced effector T cell responses.

Several molecules play key roles in maintaining the tolerogenic phenotype of LPMac. For example, IL-10 and TGF-β are major regulatory cytokines that shape gut homeostasis^17–20^, and expression of their receptors, *Il10ra*, *Il10rb*, and *Tgfbr2*, on macrophages is essential for anti-inflammatory signalling^15,16,21^. The alterations to LPMac phenotypes found in MKL1-Tg mice were likely due to a loss of their anti-inflammatory properties; LPMac from MKL1-Tg mice had lower expression of *Il10ra* and *Tgfbr2* than those from controls.

Although the evidence suggested that MKL1-Tg mice would be colitogenic, MKL1-Tg mice did not show overt symptoms of colitis, other than rectal prolapse. Nevertheless, cryptitis and infiltration of CD68^+^ cells into the submucosal layer indicated a pro-inflammatory environment in the MKL1-Tg mice. These observations indicate that MKL1-Tg mice are predisposed to develop colitis. Indeed, when MKL1-Tg mice were subjected to a DSS-induced colitis model, they showed higher susceptibility than non-Tg controls. Of note, the proximal region of the colon, which is relatively resistant to DSS-induced damage, experienced worse histopathology in the MKL1-Tg mice.

B cell infiltration or follicle formation in the colonic mucosa is one of the particular histological features of the DSS-induced colitis model^22^; although it could also reflect a reaction against epithelial irritation or local infection in the colon caused by DSS treatment^23^. DSS-induced migration of B cells into the mucosa mainly occurs relatively late after treatment^24^. To avoid excessive ablation and disruption of the mucosal layer by continuous DSS treatment, we examined the histopathology of the colon during the recovery phase of DSS-treated mice^23,24^, and observed remarkable B cell infiltration and follicle formation in the colons of MKL1-Tg mice, suggesting that MKL1-Tg mice underwent fulminant colonic inflammation.

Consistent with a previous report, we found that *MKL1* overexpression increased its nuclear localisation and promoted the formation of F-actin, both of which have been reported as prerequisite molecular events to suppress the expression of *Pparg* ^25^. The nuclear receptor PPARγ is mainly expressed in adipose tissue and the colon. PPARγ in macrophages plays a protective role against the development of inflammatory diseases, such as atherosclerosis, obesity, and colitis^12,26,27^. PPARγ is also a master regulator of macrophage M2 polarisation^12^. In this regard, we found that *MKL1* overexpression blunted M2 macrophage polarisation. In addition, we found that, even without IL-4 treatment, BMDMs from MKL1-Tg mice had compromised CD206 expression, which is consistent with previous reports that M-CSF primes M2-prone macrophages^28,29^. One of the most important functions of MKL1 is to regulate the expression of pro-inflammatory cytokines, which it accomplishes by interacting with NF-κB p65^7^. We found that, under pro-inflammatory conditions, MKL1 overexpression significantly enhanced the transcriptional activity of NF-κB p65 and boosted M1 macrophage polarisation. These observations indicate that *MKL1* orchestrates macrophage M1/M2 polarisation through interactions with other transcription factors; our results support the hypothesis that M2 macrophages protect against DSS-induced and other forms of murine experimental colitis^16,30^.

In conclusion, our study revealed the impact of MKL1 in macrophages on the development of colitis. In particular, we found that MKL1 regulates the inflammatory properties of macrophages. Our observations suggest that *MKL1* may be a potential therapeutic target for the treatment of IBD.

## Methods

### Animal experiments

C57BL/6 J mice were purchased from CLEA Japan. For the establishment of MKL1-Tg mice, a complementary DNA (cDNA) fragment encoding human MKL1 was tagged with a *FLAG* cDNA sequence at the 5′ end, then cloned downstream of a modified human *CD68* promoter element (a kind gift from Peter. J. Murray, St. Jude Children’s Research Hospital). The designed expression cassette, containing the driving promoter, IVS1, the target gene, and the BGH polyA tail, was excised using SalI and DraIII, then injected into the male pronucleus of a C57BL/6 J egg, which was implanted into a pseudo-pregnant female recipient mouse. The transgenic offspring were genotyped by PCR. For the DSS-induced colitis model, mice (8–12 weeks) received 3% DSS (160110, M.W. = 36,000–50,000, MP Biomedicals), dissolved in drinking water, for specified periods, depending on the assay. All animal care and experimental procedures were in accordance with the guidelines for the Care and Use of Laboratory Animals published by the National Research Council (The National Academy Presses, 8th edition, 2011), and were subjected to prior approval by the local animal protection authority of the Tokyo Medical and Dental University (TMDU).

### Histological and immunohistochemical analyses

We euthanised mice and excised theirs colons, which were cut open longitudinally and rinsed in 1 × PBS to remove faeces, then fixed in a Swiss roll shape in 10% Formalin Neutral Buffer Solution (Wako). Paraffin-embedded sections were stained with haematoxylin and eosin (H&E) for histological assessment. The disease severity was evaluated according to a previously described scoring system^31^. The total score was given by the sum of the scores for the proximal, middle, and distal regions of the colon. For immunohistochemical analysis, fresh colon tissues were embedded in OCT compound (Sakura Finetek). Frozen sections were fixed in 4% paraformaldehyde (PFA), blocked in Blocking One (Nacalai Tesque), and incubated with rat anti-CD68 antibody (FA-11, AbD Serotec) or rat anti-B220 antibody (RA3-6B2, BD Pharmingen), followed by incubation with Alexa Fluor® series secondary antibodies (Thermo Fisher Scientific). Samples were examined using a fluorescence microscope (Fluoview FV10i, Olympus).

### LPMac isolation and flow cytometric analysis

We cut the colons of euthanised mice into small pieces, which were serially incubated in 1 × HBSS containing 5% foetal bovine serum (FBS) and 2 mM EDTA, and Digestion Buffer containing 1 × HBSS supplemented with 5% FBS, collagenase VIII (1.5 mg/ml; C2139, Sigma-Aldrich), and DNase I (40 μg/ml; DN25, Sigma-Aldrich). The released cells were subjected to density gradient separation with 40% and 80% Percoll solutions (GE Healthcare). The cells located between the upper and lower phases were collected and stained with APC-conjugated anti-CD45 (30-F11), PE-conjugated anti-CD11b (M1/70), PE/Cy7-conjugated anti-CD11c (N418), FITC-conjugated anti-CD64 (X54-5/7.1), PE/Dazzle™ 594-conjugated anti-Ly6C (HK1.4), and APC/Cy7-conjugated anti-MHC-II (M5/114.15.2). All antibodies were purchased from BioLegend. We used a Zombie UV Fixable Viability Kit (BioLegend) to determine cell viability. Flow cytometric analysis was performed on a MoFlo™ XDP (Beckman Coulter), and the data were analysed with FlowJo® ver. 10 (Tree Star). If necessary, the cells of interest were sorted. Flow cytometric analysis was performed in the Stem Cell Laboratory, Medical Research Institute, TMDU.

### LPL isolation and ELISA

LPLs were isolated and cultured as previously described^32^. Harvested supernatants were subjected to ELISA for the determination of IFN-γ, IL-4, IL-10 (BD Biosciences), and IL-17 (R&D Systems) levels.

### BMDM preparation and M1/M2 polarisation

Femoral and tibial bone marrow cells were harvested from mice, and subsequently cultured in RPMI-1640 medium (R8758, Sigma-Aldrich) supplemented with 10% FBS, sodium pyruvate (Thermo Fisher Scientific), MEM NEAA (Thermo Fisher Scientific), and recombinant (r) M-CSF (40 ng/ml, BioLegend) for 6 days, with medium changes every 2 days. The differentiated BMDMs were treated with rIFN-γ (100 ng/ml; BioLegend) + LPS (50 ng/ml; L5273, Sigma-Aldrich) or rIL-4 (20 ng/ml; BioLegend) for 16 to 24 hours to achieve M1 or M2 polarisation, respectively. In order to evaluate M2 polarisation, we stained BMDMs treated with rIL-4 with PE-conjugated anti-CD11b (M1/70, BioLegend,), FITC-conjugated anti-F4/80 (BM8, eBioscience), and Alexa Fluor® 647-conjugated anti-CD206 (MR5D3, AbD Serotec), and analysed on a FACSCalibur™ cytometer (BD Biosciences).

### RNA isolation and quantitative RT-PCR

Cytoplasmic mRNA was isolated with a NucleoSpin® RNA Plus Kit (Takara Bio), according to the manufacturer’s instructions. We synthesised cDNA through a reverse transcription reaction using PrimeScript™ RT Reagent Kit (Takara Bio), which we then amplified with SYBR® Premix Ex Taq™ II (Takara Bio) on an iCycler iQ™ Real-Time PCR Detection System (Bio-Rad).

### Statistical analysis

Statistical comparisons were performed using unpaired two-tailed Student’s t-tests or one-way analysis of variance with post hoc Tukey’s multiple comparison tests on GraphPad Prism 7 (GraphPad Software). The results were considered statistically significant when the p-value was less than 0.05.

## Electronic supplementary material

Supplementary Information
