# Supplementary material for: MKL1 expressed in macrophages contributes to the development of murine colitis
Source: Sci Rep. 2017 Oct 20;7:13650. doi: 10.1038/s41598-017-13629-0 (PMC5651926; doi:10.1038/s41598-017-13629-0)

***MKL1* expressed in macrophages contributes to the development of murine colitis**

Jianbo An[1], Takashi Nagaishi[2], Taro Watabe[2], Taeko K. Naruse[1], Mamoru Watanabe[2], Akinori Kimura[1]

[1]Department of Molecular Pathogenesis, Medical Research Institute, Tokyo Medical and Dental University (TMDU), Tokyo, Japan

[2]Department of Gastroenterology and Hepatology, Graduate School of Medical Science, Tokyo Medical and Dental University (TMDU), Tokyo, Japan

Address Correspondence: Akinori Kimura, M.D., Ph.D., Department of Molecular Pathogenesis, Medical Research Institute, Tokyo Medical and Dental University (TMDU), 1-5-45 Yushima, Bunkyo-ku, Tokyo, 113-8510 Japan. Tel: +81-3-5803-4905, Fax: +81-3-5803-4907, Email: akitis@mri.tmd.ac.jp

Supplementary Information

Methods

RNA isolation from murine tissues

Fresh hearts, lungs, livers, spleens, kidneys, aortas, skeletal muscles, brains, and colons were harvested from mice and homogenized in TRIzol® reagent (15596018, Thermo Fisher Scientific), after which total RNA was purified, according to the manufacturer's instructions.

Immunocytochemistry staining

BMDMs were fixed in 4% PFA, permeabilised with 0.5% Triton X-100, and blocked with 1% bovine serum albumin. Cells were then incubated with goat anti-MKL1 antibody (sc-21558, Santa Cruz Biotechnology), followed by Alexa Fluor® 568-conjugated rabbit anti-goat IgG (A11079, Thermo Fisher Scientific). Slides were mounted with VECTASHIELD (H-1200, Vector Laboratories) containing 4',6-diamidino-2-phenylindole (DAPI) and visualized on a fluorescence microscope (IX73, Olympus).

F-actin staining

BMDMs were fixed in 4% PFA, permeabilised with 0.5% Triton X-100, and incubated with Alexa Fluor® 488-conjugated phalloidin (A12379, Thermo Fisher Scientific).

NF-κB p65 transcriptional activity quantification

We harvested BMDMs treated with or without LPS (100 ng/ml, 1 hour) and extracted the nuclear fractions using NE-PER™ Nuclear and Cytoplasmic Extraction Reagent (78833, Thermo Fisher Scientific). NF-κB p65 transcriptional activity was quantified in the nuclear fraction using NFκB p65 Transcription Factor Assay Kit (ab133112, Abcam), according to the manufacturer's instructions.

Supplementary figure legends

Supplementary Figure S1. C57BL/6J mice received 3% DSS or control drinking water for 4 days. We calculated (A) the proportions of $CD45^{+}CD11b^{+}CD11c^{low\ to\ mid}$ $CD64^{+}Ly6C^{+}$ LPMo, as well as the (B) absolute numbers of LPMac and LPMo. All experiments were repeated 3 times and data are shown as mean ± SD. *P<0.05.

Supplementary Figure S2. C57BL/6J mice received 3% DSS or control water for 4 days,

after which the expression level of *Mkl1* in whole colon was determined by quantitative RT-PCR (n=4 for control mice and n=5 for DSS-treated mice).

Supplementary Figure S3. C57BL/6J mice received 3% DSS or control water for 4 days, after which the expression level of *Pparg* in $CD45^{+}CD11b^{+}CD11c^{low\ to\ mid}$ $CD64^{+}Ly6C^{—}$ $MHC\text{-}II^{+}$ LPMac was determined by quantitative RT-PCR (n=3 for each group). Data are shown as mean ± SD. *P<0.05.

Supplementary Figure S4. MKL1-Tg mice overexpressed human *MKL1* in macrophages. (A) The transgenic expression cassette used to establish the MKL1-Tg mice is shown. The expression levels of total human *MKL1* and mouse *Mkl1* in (B) representative tissues and (C) $CD45^{+}CD11b^{+}Ly6C^{+}$ bone marrow monocytes and BMDMs from MKL1-Tg mice and non-Tg littermates were determined by quantitative RT-PCR (n=3 for each group). Data are shown as mean ± SD. *P<0.05.

Supplementary Figure S5. We calculated the (A) proportions of $CD45^{+}CD11b^{+}CD11c^{low\ to\ mid}$ $CD64^{+}Ly6C^{+}$ LPMo from MKL1-Tg mice and non-Tg littermates, as well as the (B) absolute numbers of LPMac and LPMo. All experiments

were repeated 3 times and data were shown as mean ± SD. *P<0.05.

Supplementary Figure S6. The expression levels of *Pparg* in $CD45^{+}CD11b^{+}CD11c^{low\ to\ mid}$ $CD64^{+}Ly6C^{-}MHC\text{-}II^{+}$ LPMac from MKL1-Tg mice and non-Tg littermates were determined by quantitative RT-PCR (n=3 for each group). Data are shown as mean ± SD. *P<0.05.

Supplementary Figure S7. Overexpression of *MKL1* affected the transcriptional activities of PPARγ and NF-κB p65. (A) Representative immunocytochemical images showing the cellular localisation of MKL1 in BMDMs from MKL1-Tg mice and non-Tg littermates. Red indicates MKL1, stained with goat anti-MKL1 antibody and Alexa Fluor® 568-conjugated rabbit anti-goat IgG. Blue indicates nuclear staining with DAPI. Scale bar indicates 10 μm. (B) We evaluated the expression of F-actin in BMDMs (histograms) from MKL1-Tg mice and non-Tg littermates by flow cytometry. F-actin was stained by Alexa Fluor® 488-conjugated phalloidin. (C) The expression levels of *Pparg* in BMDMs from MKL1-Tg mice and non-Tg littermates were determined by quantitative RT-PCR (n=3 for each group). (D) NF-κB p65 transcriptional activity was quantified in vehicle-treated and LPS (100 ng/ml, 1 hour)-treated BMDMs from MKL1-Tg mice and

non-Tg littermates (n=3 for each group). Data are shown as mean ± SD. *$P<0.05$.

Supplementary Figure S8. We evaluated the expression of CD206 in $CD11b^{+}F4/80^{+}$ BMDMs (histograms) from MKL1-Tg mice and non-Tg littermates by flow cytometry. We calculated and plotted the MFI. Experiments were repeated 3 times and data are shown as mean ± SD. *$P<0.05$.

Supplementary Figure S9. (A) Representative images of whole colon from female MKL1-Tg mice and control littermates at 8 weeks of age. (B) Representative histological images (high-power field) of the proximal region of the colon stained with H&E.

Supplementary Figure S10. We calculated the (A) proportions of $CD45^{+}CD11b^{+}CD11c^{low\ to\ mid}$ $CD64^{+}Ly6C^{+}$ LPMo from MKL1-Tg mice and non-Tg littermates, as well as the (B) absolute numbers of LPMac and LPMo. All experiments were repeated 3 times and data are shown as mean ± SD. *$P<0.05$.

Supplementary Figure S11. (A) The modified protocol to study the recovery phase of DSS-induced colitis. After the administration of 3% DSS for 5 days, the drinking solution

was replaced with regular water for an additional 3 days, then mice were euthanized for assessment. (B) Representative macroscopic features of the recovery phase-colons from DSS-treated MKL1-Tg mice and control littermates. (C) Representative immunohistochemical images overlaid with phase contrast images showing follicle-forming B cells (red) in the colonic tissues of controls (left) and MKL1-Tg mice (right) during the recovery phase of DSS-induced colitis. Specimens were stained with rat anti-B220 antibody and Alexa Fluor® 594-conjugated goat anti-rat IgG.

Supplementary Fig. S1

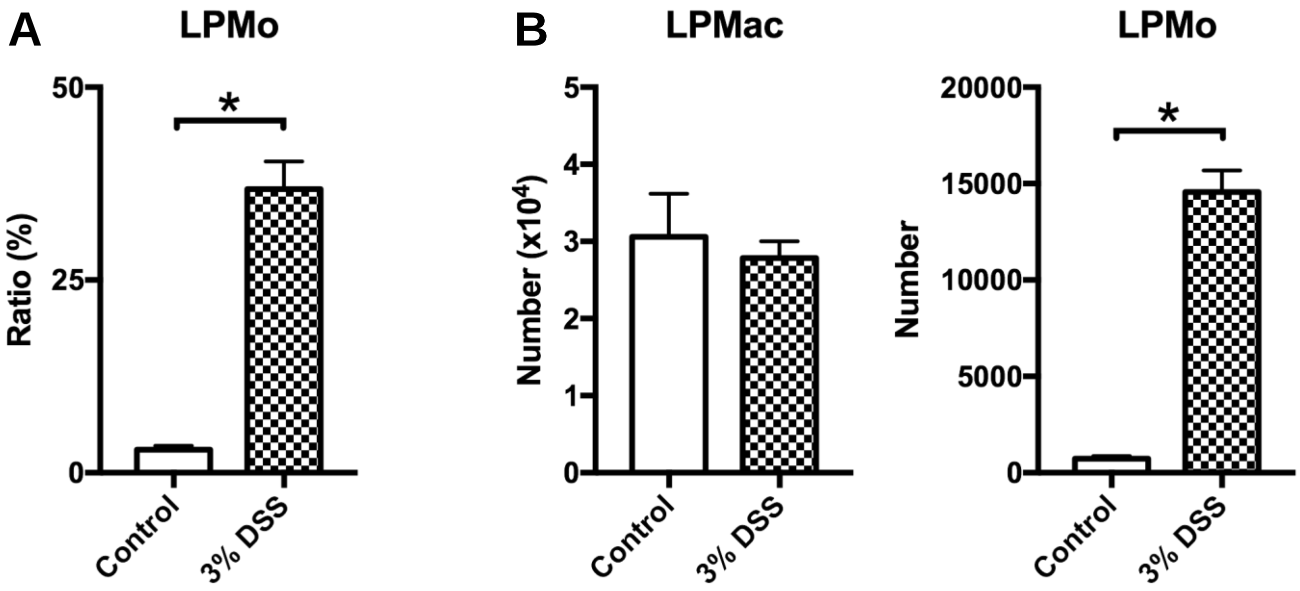

Supplementary Fig. S2

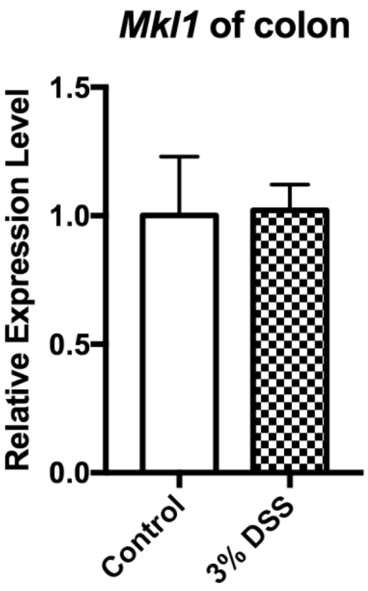

Supplementary Fig. S3

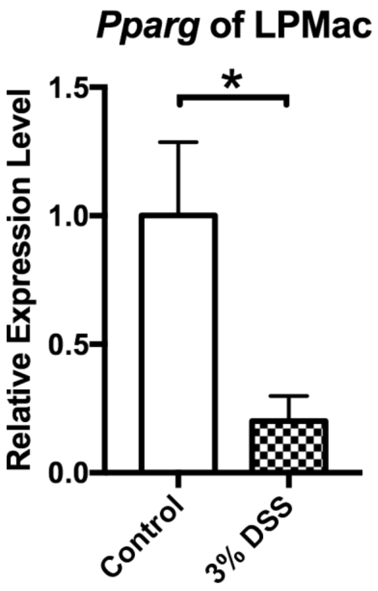

Supplementary Fig. S4

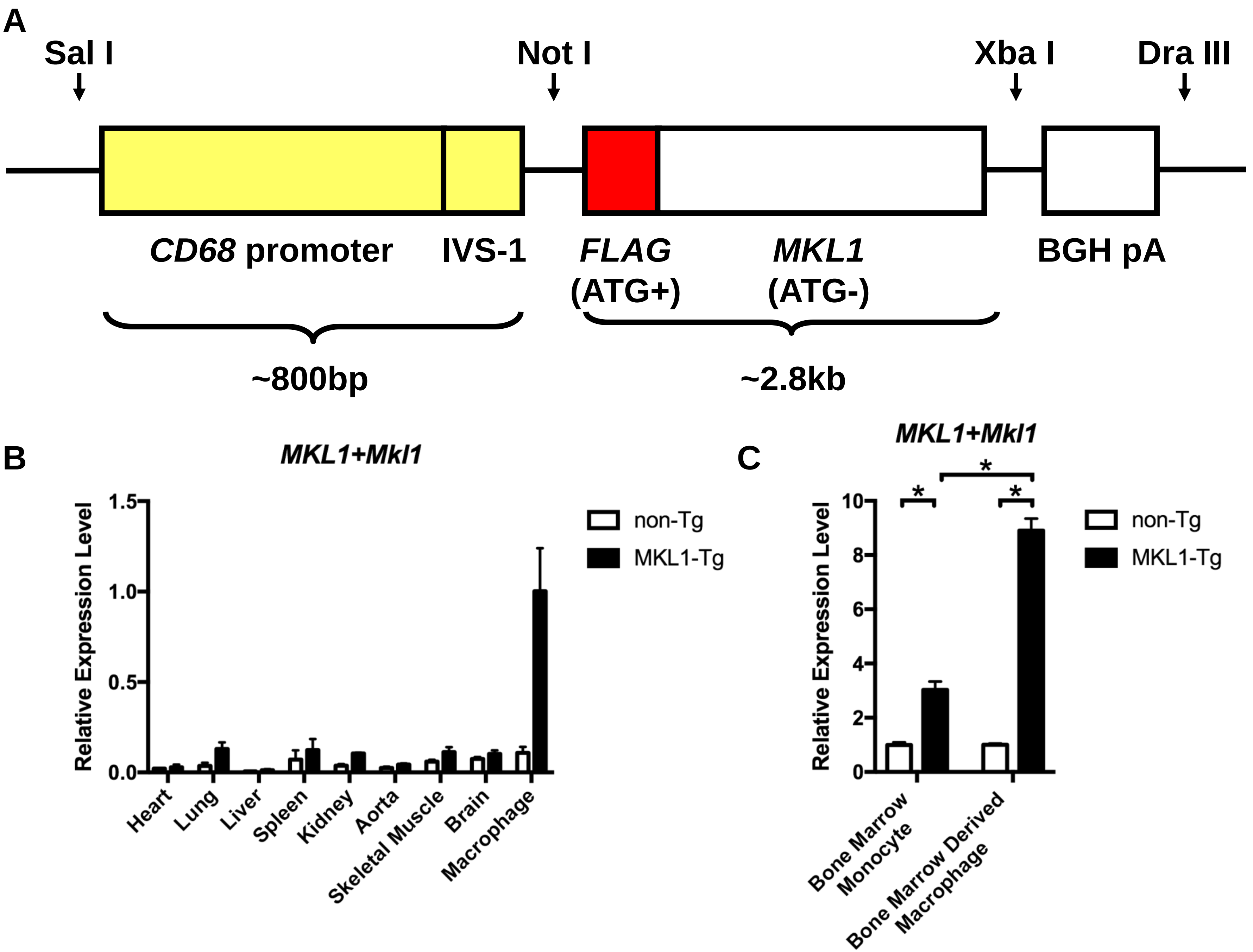

Supplementary Fig. S5

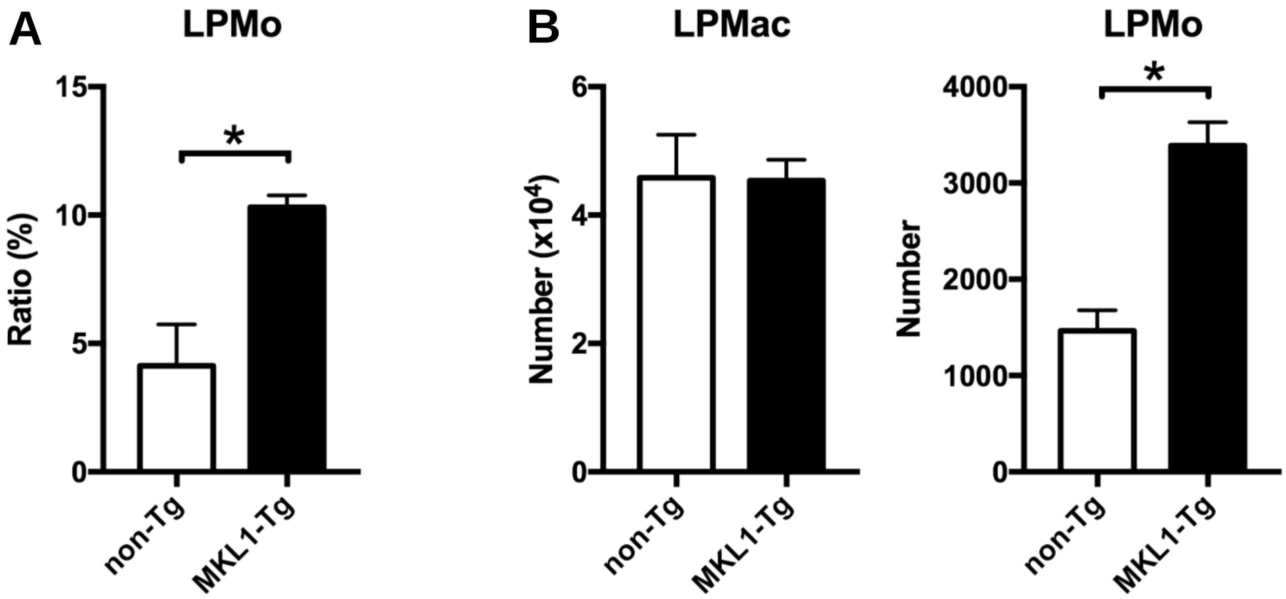

Supplementary Fig. S6

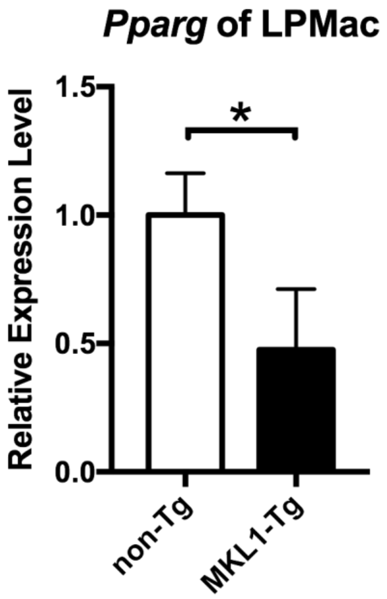

Supplementary Fig. S7

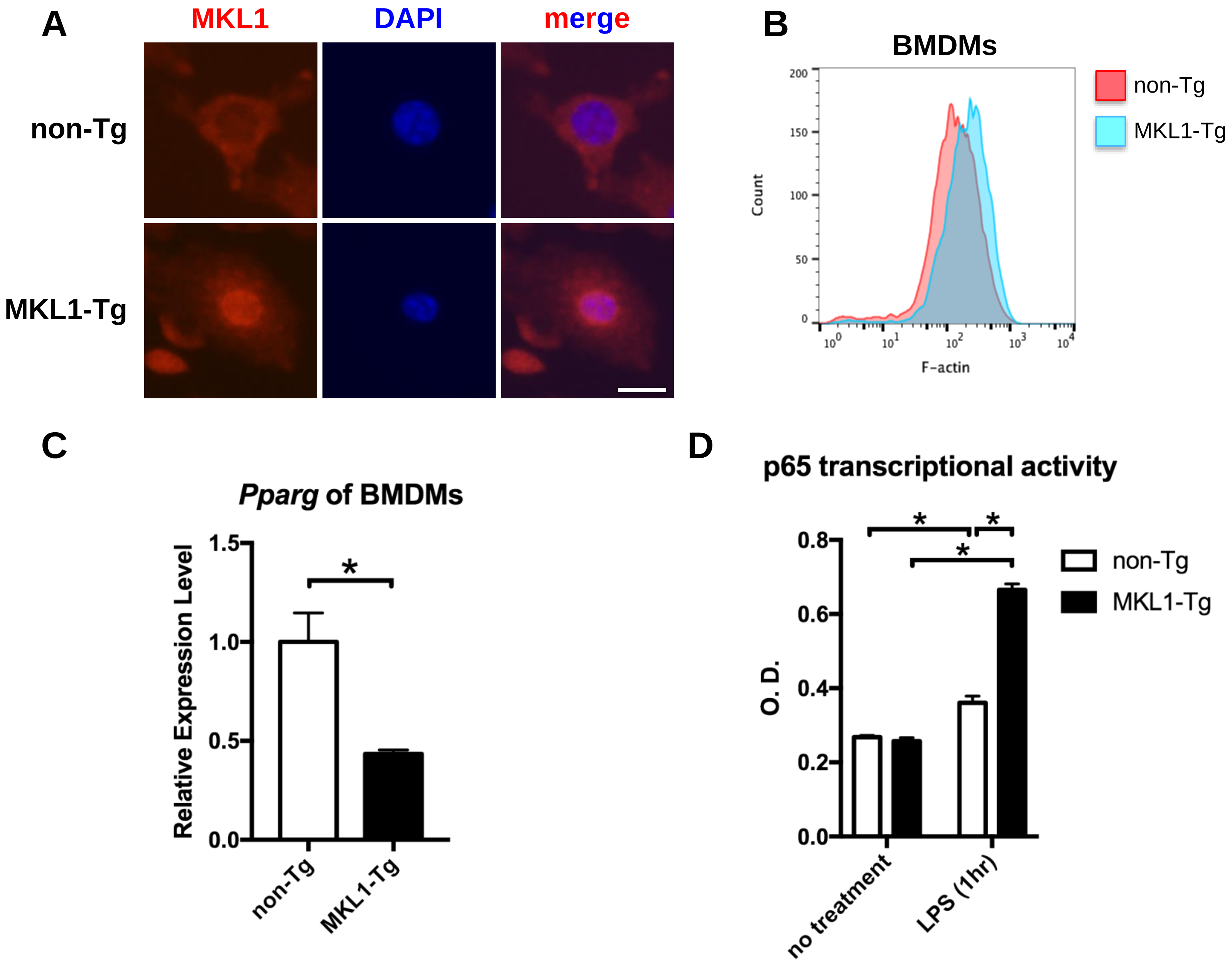

Supplementary Fig. S8

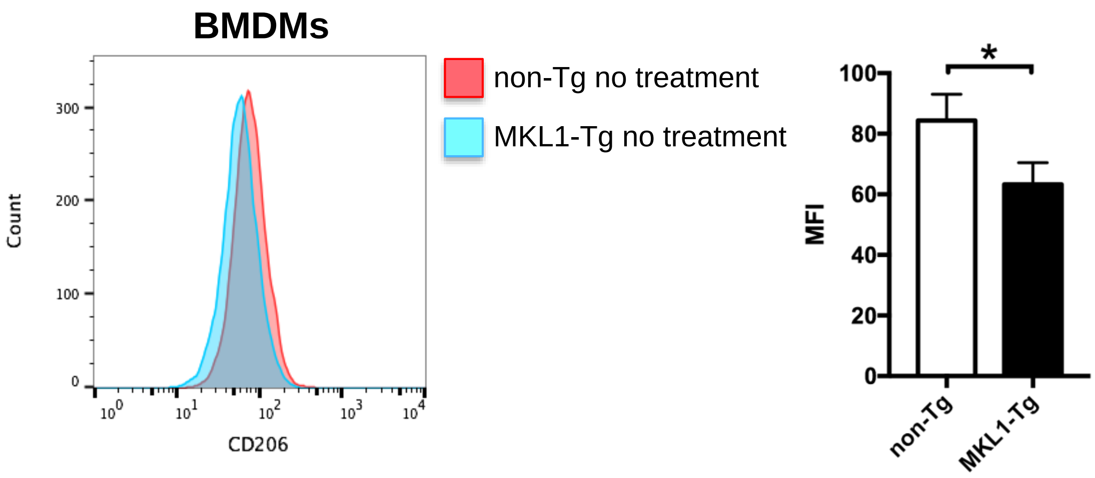

Supplementary Fig. S9

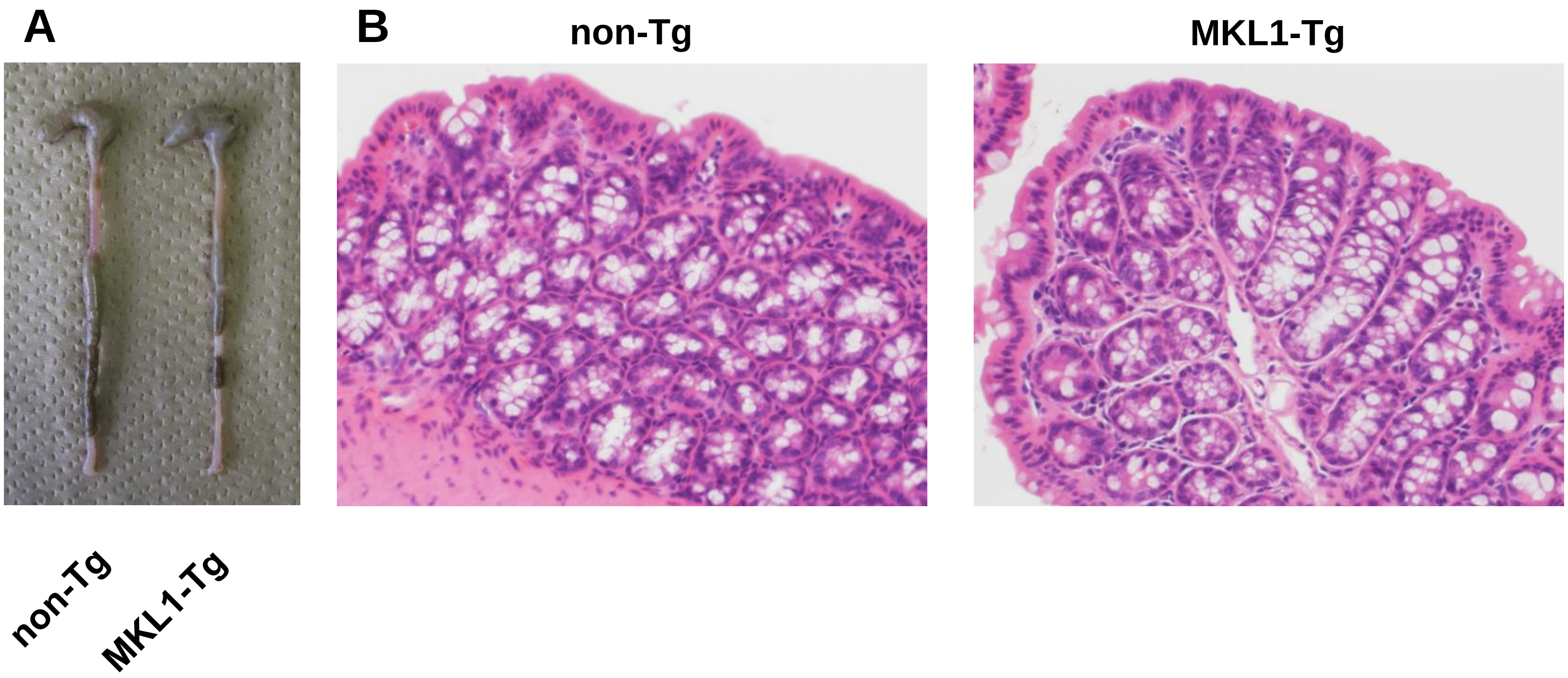

Supplementary Fig. S10

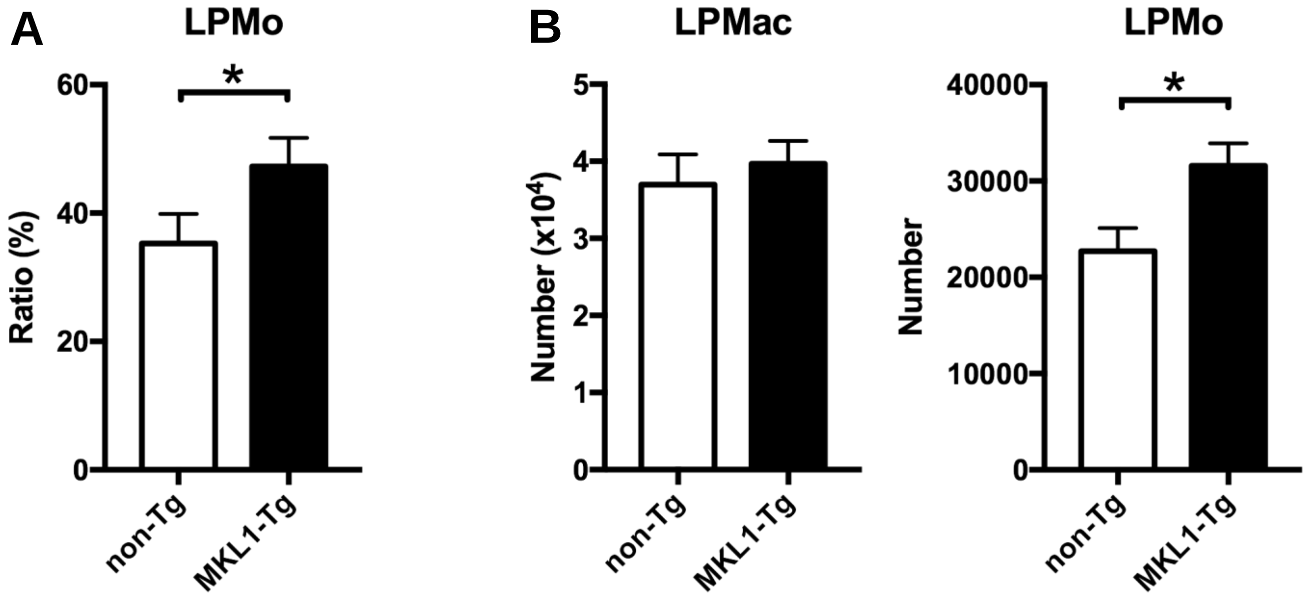

Supplementary Fig. S11

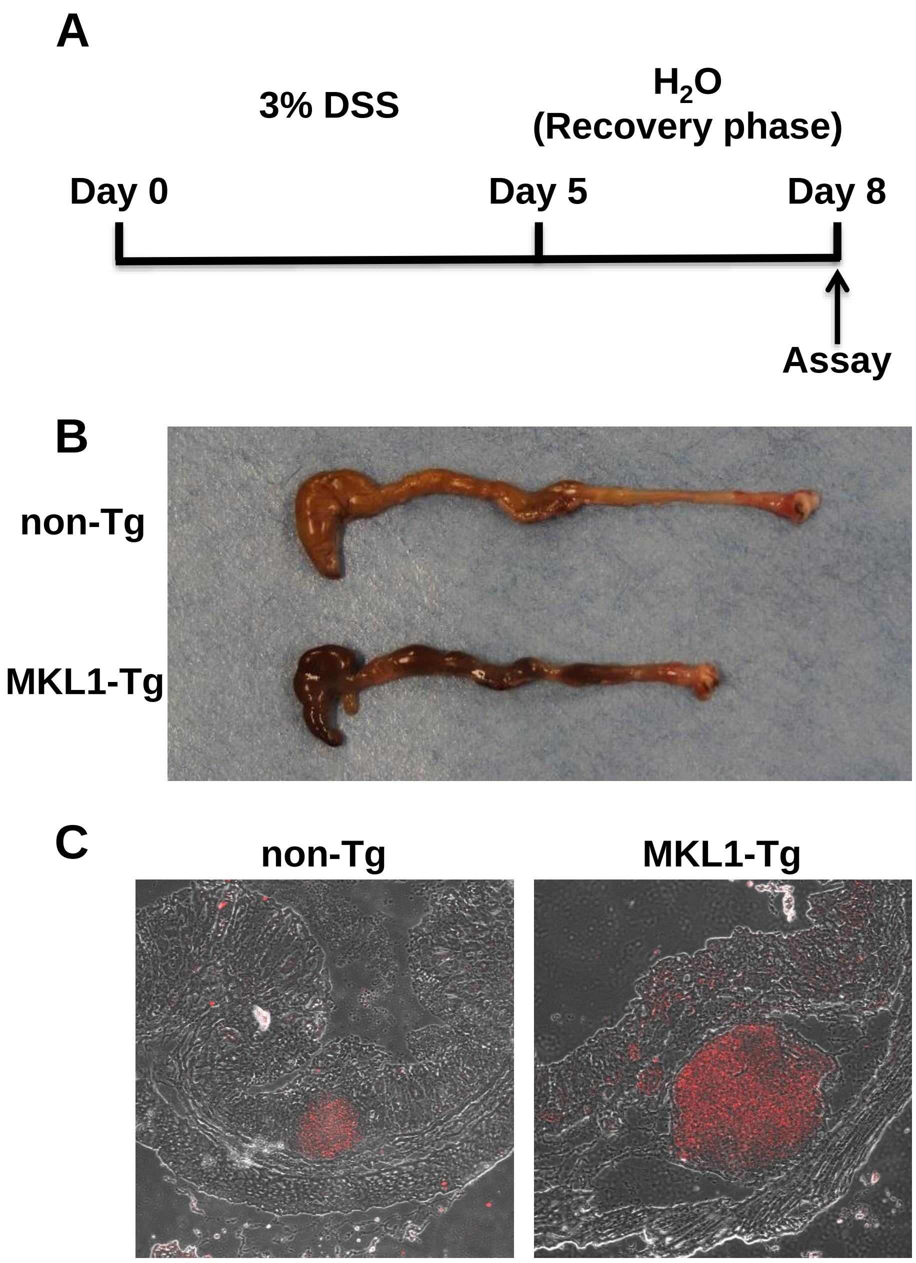

Supplement: Supplementary file 1 — Supplementary Information [file 41598_2017_13629_MOESM1_ESM.pdf]
